# Application of Immersive Virtual Reality for Assessment and Intervention in Psychosis: A Systematic Review

**DOI:** 10.3390/brainsci13030471

**Published:** 2023-03-10

**Authors:** Karen Chui-Shan Chan, Christy Lai-Ming Hui, Yi-Nam Suen, Edwin Ho-Ming Lee, Wing-Chung Chang, Sherry Kit-Wa Chan, Eric Yu-Hai Chen

**Affiliations:** 1 Department of Psychiatry, School of Clinical Medicine, Li Ka Shing Faculty of Medicine, University of Hong Kong, Hong Kong SAR, China; 2 State Key Laboratory of Brain and Cognitive Sciences, University of Hong Kong, Hong Kong SAR, China

**Keywords:** VR, virtual reality, immersive, psychosis, assessment, intervention, digital technologies, systematic review

## Abstract

Virtual reality (VR) has emerged as a safe and non-invasive technology for the assessment of psychotic symptoms, social and cognitive impairments, and psychosocial intervention in improving outcomes in psychosis. This study systematically reviewed the current state of evidence in applying semi- and fully immersive VR for assessing and treating patients with psychosis. A systematic review was conducted adhering to the PRISMA statement and was conducted in Embase, PsycINFO, and PubMed databases for articles published between January 2013 and April 2022, which identified 28 eligible studies, including 12 for assessment and 16 for intervention. In the assessment studies, not all VR tasks could distinguish the differences between patients and healthy controls regarding their physiological responses, paranoid ideation, and certain aspects of cognitive functioning such as memory bias on the object tasks. Comparatively, VR-based interventions are more promising, especially for improving cognitive impairments, social skills, agoraphobic avoidance, negative and positive affective states, auditory verbal hallucination, paranoid ideation and persecutory delusions, and other psychiatric symptoms in patients. We conclude that more rigorous studies are needed to confirm treatment effectiveness and to understand the underlying mechanism of VR-based intervention for psychotic disorders. Future studies should also improve the reliability and validity of VR-based assessments for psychotic disorders.

## 1. Introduction

Patients with psychotic disorders including schizophrenia exhibit symptoms such as delusion, hallucination, and thought disorder. The conditions usually come with adverse consequences on one’s social and cognitive functioning, as well as positive and negative symptoms [1,2]. Psychosis is still not easy to diagnose and treat based on our latest understanding of its etiology [3]. Thus, researchers and clinicians increasingly feel an urge to apply the latest technology to explore new alternatives for diagnosing and treating patients with psychosis [4,5].

Virtual reality (VR) is regarded as a non-invasive and safe technology that provides a fully digital, three-dimensional (3D) experimental experience to the user. The features of VR technology include immersiveness, control, and flexibility, which potentially make it a valid and effective tool for assessing and treating patients with psychotic disorders [6]. The illusion of reality in the user’s senses has been shown to induce similar physical and psychological reactions in real life [7]. This could allow researchers and clinicians to access patients’ cognition, emotions, and verbal and behavioral interactions with a pre-set stimulus in real-time VR environments. Unlike conventional assessments, which are usually conducted under controlled settings in the laboratory or clinic, VR provides patients with a more ecologically valid, real-life setting through interactive and immersive experiences [8].

VR also offers the control to tailor-make a social encounter or daily life situation that is not easy to replicate in the real world. This could help to personalize interventions to align with each’s heterogeneous needs, which is particularly useful for psychosis patients who usually manifest heterogeneity in symptom presentation, outcomes, and treatment response. In a virtual environment, these patients feel safe to explore and practice new skills until they can master and then transfer what they have learned into similar real-life situations. While VR allows for replicating real-life situations, the same VR scenario could also be presented uniformly to many patients with their different reactions being recorded, thereby greatly increasing comparability across studies [9].

Another key advantage that VR offers is flexibility, as users can enter the VR environment anytime and anywhere. Some psychosis patients with severe depressive or anxiety symptoms, or patients living in remote areas, could still access the intervention at home as long as a head-mounted display (HMD) is provided to them [8]. VR could also offer a greater degree of privacy, facilitate self-training, and lower the demand for therapists’ time [10,11]. Moreover, some VR technologies offer interactive components such as a virtual 360-degree landscape and the provision of immediate feedback, making the application even more appealing and entertaining [12]. Technological advances continue to drive the development of VR, which is expected to improve in its functions, including in the experience of immersiveness. Such advancement may also bring down the costs of VR-related equipment, which will make its application more widely acceptable and economical.

The interest in applying VR technology to patients with psychotic disorders has been strong, as reflected in the rising number of published articles [13]. In general, VR simulations can be classified into three categories: non-immersive, semi-immersive, and fully immersive. Non-immersive simulations are conventional computer-generated 3D environments that the users view from a computer monitor. Semi-immersive VR provides users the experience of a partially virtual environment with a digital image mixed with the physical environment. Fully immersive VR gives users the most realistic and vivid experience of virtual worlds, with which they can interact by using all of their senses (except taste). In more advanced headsets, the physiological and behavioral responses of the users can be tracked and recorded via tactile gloves, body motion detection, or built-in cameras, etc.

In this systematic review, we mainly focus on the use of semi- and fully-immersive VR, as it is proven that the feeling of presence is a crucial element in enhancing user engagement [14]; this is in contrast to previous systematic reviews on virtual reality in psychosis, which included both non-immersive/3D computer-screen-based as well as immersive VR [6,15,16]. Moreover, these previous reviews had slightly different patient groups of interest that also included mood disorders [15] or examined the validity of VR-based assessment only in the domain of social functioning [16]. We conducted a systematic review on the studies that applied immersive VR technology in patients with psychotic disorders. Specifically, we summarized the current evidence (1) to examine the validity of using VR as an assessment tool in different aspects of disorders such as their psychopathology or social and cognitive functioning as well as (2) to evaluate the effectiveness of using VR as a psychosocial intervention for treatment in psychotic disorders. We also provide practical recommendations on VR application for researchers and clinicians that can guide future research and clinical applications.

## 2. Materials and Methods

### 2.1. Information Sources and Search Terms

A literature search was executed in three electronic databases: Embase, PsycINFO, and PubMed. The systematic review was conducted following the PRISMA statement. The period of published articles was restricted to the past ten years between January 2013 and April 2022. Searches were limited to peer-reviewed articles that were written in the English language. The keywords used in the search criteria were as follows: (Virtual Reality OR VR) AND (Psychosis OR Schizophrenia OR Severe Mental Illness). Duplicate studies were removed in the initial screening. The titles were then read to identify relevant studies. After that, the abstracts and full texts were reviewed to determine the eligibility of the studies. One author, K.C., conducted the screening process in consultation with C.H., and any ambiguities were resolved through discussion.

### 2.2. Eligibility and Inclusion Criteria

The following inclusion criteria were applied: (1) empirical studies employing either a randomized controlled trial (RCT), non-randomized trial, pilot study, or single-arm study; (2) experimental groups with participants that had been diagnosed with schizophrenia or non-affective psychosis (schizophreniform disorder, schizoaffective disorder, brief psychotic disorder, or psychosis not otherwise specified); (3) application of VR for assessment or intervention in psychotic symptoms, cognition, social skills, stress management, or daily functioning; and (4) the adoption of immersive VR using HMD or 3D-polarized glasses.

### 2.3. Data Extraction

The relevant data were extracted from each of the studies and summarized into a table, with the key variables as follows: author, country of origin, study design, population, VR equipment, assessment task or intervention tool, session, baseline and outcome measures, and key findings. The process was conducted independently by K.C., consulting with C.H. to resolve uncertainty.

### 2.4. Risk of Bias in Individual Articles

All the eligible studies were subjected to a risk-of-bias assessment. For RCT, crossover trials, and pilot-randomized comparative trials, the revised Cochrane risk-of-bias tool for randomized trials (version 2) (RoB 2) [17] was used. For the rest of the included papers, the ROBINS-I tool was used to assess the risk of bias in non-randomized studies [18]. A quality rating was conducted independently by K.C., consulting with C.H. to resolve uncertainty. Supplementary Material S1 shows the results of the risk-of-bias assessment.

## 3. Results

### 3.1. Search Results and Summary of the Studies

**Figure 1:**
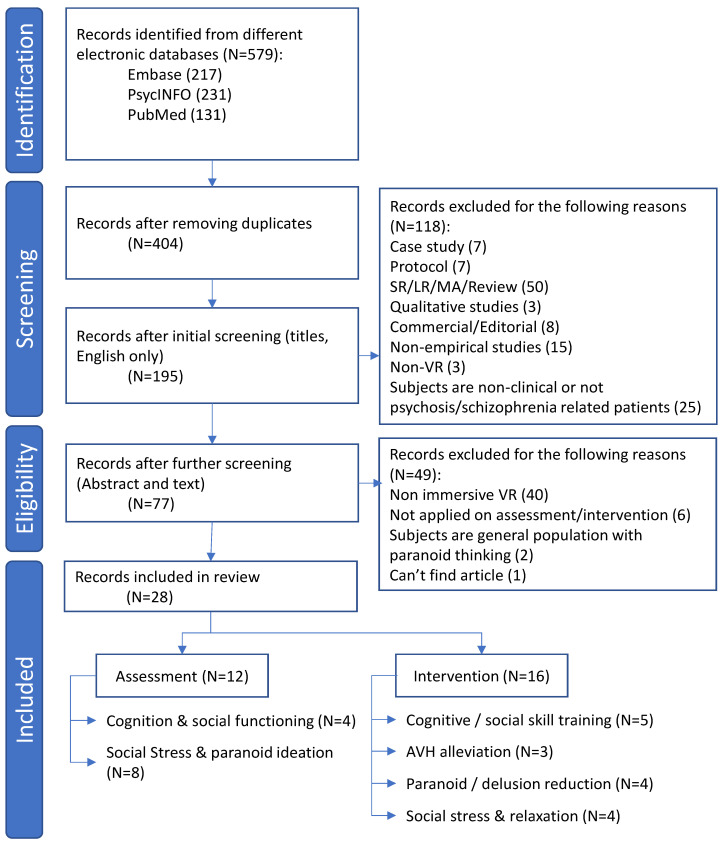
PRISMA (Preferred Reporting Items for Systematic Reviews and Meta-Analyses) flow diagram. Abbreviations: AVH, auditory voice hallucination; LR, literature review; MA, meta-analysis; SR, systematic review; VR, virtual reality.

Figure 1 shows the PRISMA flow diagram of the search [19]. A total of 579 studies were identified from the initial literature search. After removing the duplicates, 404 publications were found to be potentially relevant. The studies were then further screened by reading their titles, with 195 articles being left. By executing the abstract and full-text analysis, 77 studies were qualified as empirical quantitative studies including participants that had been diagnosed with any psychotic disorders. Upon focusing on the application of immersive VR for assessment and intervention in psychosis, a final 28 studies were eligible for our review. The most common reasons for excluding studies were the use of non-immersive VR (40 studies) and the application of VR outside of assessment and intervention (6 studies).

Among these 28 studies, there were 12 papers related to the application of VR for assessment and 16 papers related to the same for intervention (Table 1). Of those assessment studies, the focus of the measurement could be classified into two areas: cognitive and social functioning (N = 4) and social stress and paranoid ideation (N = 8). It was noted that seven papers on the assessment of paranoid ideation were conducted by the same research team based in the Netherlands [20], of which, six used the same group of subjects but with different topic focuses [13,21,22,23,24,25].

Of those 16 intervention studies, the target of the treatment could be classified into four areas: cognitive and social skills training (N = 5), auditory verbal hallucination (AVH) alleviation (N = 3), paranoid/delusional ideation (N = 4), and social stress and relaxation (N = 4). Of the five papers on social skills training, two shared the same sample pool [26,27]. Similarly, two studies on paranoid delusion shared the same sample pool [28,29].

**Table 1:** Studies on VR-based assessment and interventions.

Authors/Year/Country	Study Design	Study Population	VR Equipment	Interventions	Sessions	Baseline/Outcome Measures	Key Findings
**Assessment: cognition & social functioning**							
Dietrichkeit et al., 2020 [30] Germany	Pilot study	39 PP (M 34.72, SD 8.68, male 62%) and 20 HC (M 30.55, SD 8.54, male 50%); N = 59	HMD (Oculus Rift DK2)	Two VR scenes: a street and a metro station (social tasks)/a beach and a campground (object tasks). Half of the subjects provided feedback on the recollection task.	4 (baseline assessment, one task each and a post-diagnostic assessment)	MINI, PANSS, BCIS, VR task performance (number of correct recollections)	Patients reported comparable performance on the object task, with only the social tasks showing lower performance. Feedback had little effect on patients’ cognitive insight.
Han et al., 2014 [31] Korea	Pilot study	23 SZ (M 28.9, SD 3.4, male 43%) and 22 HC (M 27, SD 3.6, male 41%); N = 45	HMD (eMagin Z800 3DVisor) with a head tracker and an eye tracker	Conversations with two avatars sitting at the same table with the participants in four scenarios, each of which contained both listening and speaking components.	1	RPM, TMT-B, PANSS, PQ (after the experiment) and VREQ (after the task)	Patients had active avoidance of eye contact during three-party conversations. Patients took longer pauses before speaking, while both subject groups demonstrated a longer time to begin speaking in unpleasant scenarios. Useful measurement tool for social behaviors.
Miskowiak et al., 2022 [32] Denmark	Validation study	40 MD (M 35.3, SD 11.7, male 40%), 41 PSD (M 24.2, SD 3.9, male 58.5%), and 40 HC (M 30.4, SD 9.6, male 35%); N = 121	Standalone HMD (Oculus Go 32 GB, LCD display of 5.5 inches, 1280 × 1440, 72hz refresh rate) & hand-held controller	CAVIR measures subjects’ verbal memory, processing speed, executive functioning, attention and working memory through five tasks performed in a simulated kitchen to make a meal.	1 (self-administrated with a 15 min. duration)	OTS, SWM, RVP, RAVLT, WAIS-III, RBANS Coding, DS, VFT, TMT-A&B, DART, FAST, UPSA-B, PQ, VRSSQ, CAVIR, HDRS-17 + YMRS for MD/HC; SAPS + SANS for PSD	CAVIR is a sensitive and valid tool for assessing cognitive impairments in MD and PSD that occur in daily life. The results of the CAVIR and the neuropsychological tests showed strong correlations. CAVIR is also cost-effective due to its self-administration and usage of inexpensive equipment (Oculus Go; priced at $200).
Souto et al., 2013 [33] Portugal	Pilot study	12 SZ (M 36.25, SD 5.754, male 75%) and 12 HC (M 36.25, SD 5.754, male 75%); N = 24	A screen (4 × 3 m), 2 multimedia projectors with passive polarized glasses, and a surround-sound system	RV-REF (3D avatars and virtual reality environment) with a task to identify the six fundamental facial expressions, including sadness, happiness, fear, anger, disgust, and surprise.	1 (25 min, including putting in and taking out electrodes)	MMSE, GAF, PSP, RV-REF (correct and error data), EEG (F3 and F4)	While SZ patients showed lower scores, the difference was not statistically significant. Happiness and anger received higher accuracy, and major difficulties came from fear and disgust recognition for both groups. The anger and disgust stimuli caused alterations in patients’ alpha frontal activity.
**Assessment: paranoid ideation**							
Counotte et al., 2017 [21] Netherlands	Cross-sectional, between-subjects	44 ROP (M27, male 79.5%), 17 UHR (M22, male 41.2%), 37 SPP (M27, male 59.5%), and 45 HC (M 24, male 46.7%); N = 170 (143 HR available)	Vizard software (CleVR), Logitech F310 Gamepad, Sony HMD headset model HMZ-T1, 1280 × 720, 51.6 FOV, and a 3DOF tracker	Virtual bar with 3 stressors (avatar density/ethnic density/hostility): (1) No stress (6 avatars, >80% Dutch, neutral expression); (2) 40 avatars; (3) 40 majority non-self ethnicity; (4) 40 angry/hostile expression; (5) 3 stressors.	1 (exposed to VR-induced social stress in 5 settings lasting 4 min each)	Before and after/during VR task: SSQ, HR, SCL	Heart rate, HF, LF/HF, and skin conductance level were all significantly impacted by the quantity of virtual social stressors in all groups. Instead of higher autonomic responsiveness to social stresses, psychosis liability was associated with lower parasympathetic activity in virtual social contexts, which implies generally high levels of arousal.
Geraets et al., 2018 [22] Netherlands	Cross-sectional, between-subjects	50 ROP (M26, SD 4.6, male 80%), 19 UHR (M24.3, SD 4.4, male 36.8%), 40 SPP (M26.5, SD 4.8, male 55%), and 47 HC (M 24.3, SD 4.3, male 46.8%); N = 170 (156 completed data)	Vizard software (CleVR), Logitech F310 Gamepad, Sony HMD headset model HMZ-T1, 1280 × 720, 51.6 FOV, and a 3DOF tracker	Five virtual café visits with various social stressors (crowdedness, ethnicity, and hostility)	1 (each VR visit lasted 4 min with a 5 min break between experimental blocks)	Baseline: CAARMS, SUD, SOFAS, SERS. During VR tasks: IPD by the VR software. Outcome: SSPS	When social stresses were given in the VR settings, interpersonal distance (IPD) widened. IPD regulation between the groups did not differ. IPD may be impacted by more general states such as psychological stress, which are common but not unique to psychosis.
Hesse et al., 2017 [34] Germany	Randomized, controlled cross-over	26 PP (M34.52, SD 8.7, male 71%) and 20 HC (M 32.3, SD 8.5, male 65%); N = 46 (including 5 drop-outs from PP)	HMD and Gamepad were used without further details	An open-plan office was simulated in VR. Participants were asked to complete two tasks with two types of social feedback—co-operative or rejective—from 5 avatar colleagues.	3 (one for clinical interview, trait, and questionnaire and two for VR tasks) weekly	PANSS-DS, PSYRATS-DS, CDSS, SSPS, IPQ, GAF. Neurocognition: VLMT (learning/recall), TMT-A/B, MWT-B	PP and HC started with large baseline differences. Statistical trends indicated that social rejection increased paranoid ideations for PP. Additionally, PP displayed a greater sense of presence than HC. High convergent validity was demonstrated between state assessments of paranoid ideations and traditional delusion measures.
Jongeneel et al., 2018 [23] Netherlands	Cross-sectional, between-subjects	94 HC + SPP (M 25.4, SD 4.6, male 51%) and 75 UHR + ROP (M 25.4, SD 4.7, male 65.3%); N = 169	CleVR software; Logitech Chillstream Gamepad, HMD (Emagin Z800 3D Visor) featuring SVGA 800 × 600 24 bit, 40 FOV, and a 3DOF tracker	Five virtual café visits with various social stressors (crowdedness, ethnicity, and hostility)	1 (five experiments of 4 min each)	CAARMS, SOFAS for UHR, SERS, SSPS, SUD	Negative self-esteem, rather than positive self-esteem, was linked to the subjects’ momentary paranoia, peak subjective distress, or reactivity to social stressors. All subject groups reported experiencing more paranoia as the quantity of social stressors rose. Negative self-esteem had a greater effect on stress reactivity in those with lower psychosis liability than in those with higher liability.
Pot-Kolder et al., 2017 [24] Netherlands	Cross-sectional, between-subjects	55 ROP (M 26, SD 5, male 76.4%), 20 UHR (M 24 SD 4 Male 35%), 42 SPP (M 26, SD 5, male 54.8%), and 53 HC (M 25, SD 4, male 47.2%); N = 170	Same as Jongeneel et al., 2018 [21]			CAARMS, SOFAS for UHR, CASH, SCAN, DACOBS, DOG, GPTS. Outcome: SSPS	Higher intensity of (1) cognitive biases attention to threat, (2) belief inflexibility, and (3) external attribution was linked to higher psychosis liability. The number of cognitive biases present was correlated with an increase in paranoid response. Further, attention to threat bias and external attribution bias both strengthened the impact of social environmental stressors on paranoid ideation.
Veling et al., 2014 [20] Netherlands	Pilot study	17 FEP (M 27.3, SD 5.5, male 82.4%) and 24 HC (M 29, SD 9.2, male 83.3%); N = 41	CleVR software; Logitech Chillstream Gamepad, HMD (Emagin Z800 3D Visor) SVGA 800 × 600 24 bit, 40 FOV, and built-in 3DOF tracker	In a VR café, subjects were asked to locate the numbered avatars & memorize the number under four scenarios: 2 ethnic appearances (own or other) × 2 population density (low/high) of avatars.	1 (3.5 min for HC and 4 min for FEP for each task × 4)	Baseline: SIAS, GPTS, DACOBS, SSQ, SERS. During VR: GSR, HR, SSPS. Outcome: SSQ, IPQ	In comparison to HC, FEP showed considerably more paranoid thoughts, cognitive biases, more subjective distress, and poorer self-esteem. For both groups, there was a significant correlation between paranoid thoughts regarding avatars in VR and paranoia in the real world. Galvanic skin reaction was noticeably stronger in VR with avatars of other ethnicities in FEP but not in HC.
Veling et al., 2016a [25] Netherlands	Cross-sectional, between-subjects	75 ROP + UHR (M 25.4, male 65.3%) and 95 SPP + HC (M 25.4, male 50.5%); N = 170	CleVR software, Logitech F310 Gamepad, HMD (Sony HMZ-T1) featuring 1280 × 720, 51.6 FOV, and a 3DOF tracker	Same as Jongeneel et al., 2018 [21]		SIAS, GPTS, CTQ-SF, CAPE, SUD, SSPS	When the total number of virtual environmental stressors rose, the effects of childhood trauma on paranoia and subjective distress were noticeably larger across all subjects. The impact of childhood trauma on peak subjective distress and stress reactivity during experiments was amplified by higher psychosis liability.
Veling et al., 2016b [13] Netherlands	Cross-sectional, between-subjects	55 ROP (M 26, SD 4.7, male 76.4%), 20 UHR (M 24, SD 4.5, male 35%), 42 SPP (M 26.4, SD 4.8, male 54.8%), and 53 HC (M 24.6, SD 4.4, male 47.2%); N = 170	Same as Veling et al., 2016a [23]			CAPE, GPTS, SSPS, SIAS, SUD	Along with increasing levels of social stress in the environment, paranoia and subjective distress rose. Population density had a significant positive impact on both paranoia and distress. Subject distress was not significantly correlated with hostility, but paranoia was. There was no correlation between paranoia and distress and ethnic density. The degree of paranoia and distress in response to social stress was positively impacted by psychosis liability and pre-existing symptoms.
**Intervention: cognitive/social skill training**							
La Paglia et al., 2013 [35] Italy	Non-randomized CT, pilot study	6 VR-CRT (M 31, SD 14.6) and 6 IPT (M 35, SD 9.9); N = 12 SZ	Neuro-VR vers. 2.0 software, no details on HMD	Virtual reality environment for daily life tasks; the VR attention training program involved hierarchical task sequences arranged in 4 different VR environments (park, valley, beach, and supermarket)	10 (VR-CRT weekly individual sessions with each lasting ~90 min. IPT was in-group on a weekly basis, 60 min)	Pre–post measures: MMSE, FAB, TMT, ToL, WCST, and SCWT	Significant improvement in the assessed cognitive dimensions by VR training. VR training and IPT both led to better results on activities requiring divided attention. VR training was linked to fewer cognitive deficiencies and better planning.
La Paglia et al., 2016 [36] Italy	Non-randomized CT, pilot study	9 VR-CRT (M 29, SD 12.05, male 66.6%) and 6 IPT (M 35, SD 9.9, male 100%); N = 15 SZ	Neuro-VR vers. 2.0 software, no details on HMD	Same as La Pagilia et al., 2013 [33]		Pre–post measures: MMSE, ToL, TMT (A/B), FAB, WCST	VR training and IPT both led to better performance on sustained attention tasks. VT training was also linked to gains in divided attention and planning.
Rus-Calafell et al., 2013 [26] Spain	Single arm, uncontrolled pilot study	12 SZ + SZA (M 36.5, SD 6.01, male 58.3%); N = 12	A laptop, 3D glasses, and headphones.	Soskitrain: patients practiced social interactions with virtual avatars in everyday settings (e.g. a supermarket or bar) and experienced progressive learning in the social skills repertoire.	16 (twice weekly over eight weeks, individual basis with each session lasting ~60 min)	Baseline: SCIP, WMT, VLT-I/II, VFT, PST, CPT II. Outcome: SUS	Post-treatment results showed strong presence sense and good verisimilability of the virtual environment. Acceptability scores were similarly high. Between deficiencies in verbal learning and processing speed and sensation of presence, a substantial negative association was discovered.
Rus-Calafell et al., 2014 [27] Spain	Single arm, uncontrolled pilot study with 4- month follow-up	12 SZ + SZA (M 36.5, SD 6.01, male 58.3%); N = 12 (3 dropouts)	Same as Rus-Calafell et al. 2013 [24]			PANSS, AI, SSIT, SADS, SFS, qualitative and VR acceptance assessment	Positive symptoms, psychopathology, social anxiety and discomfort, avoidance, and social skill mastery significantly improved in the participants. The patient’s ability to apply new abilities to daily tasks was aided by the VR training. FU: 4 months; all gains maintained
Vass et al., 2021 [37] Hungary	Randomized pilot study with 3-month follow-up	9 VR-ToMIS (M 38.6, SD 13.49, male 55.5%) and 8 VR (M 48.8, SD 8.87, male 37.5%); N = 21 SZ (4 dropouts)	Samsung’s Gear VR, a Samsung S7 smartphone, and a Samsung Simple Controller. Environment (vTime) and avatar by Temporal Disc Controller (TDC)	VR-ToMIS: Each session had 3 consecutive steps: (1) simulated social interactions with an avatar for a conversation, (2) a task to visualize the inferred emotions of the avatar, and (3) discussion with a therapist	9 (weekly individual sessions of 50 min; 8 VR sessions and one preliminary pre-briefing session)	PANSS, RBANS, WCST-64, faux pas test and cartoon stories task, BCMET, the Hungarian metaphor & irony test, LQoLP, SSQ	Positive symptoms, one neurocognitive domain (immediate memory), ToM, and pragmatic language abilities all improved as a result of the VR training, but there was no appreciable increase in quality of life. Modest to significant therapeutic effects were linked to significant alterations in the VR-ToMIS group. FU: 3-month (no data was shown)
**Intervention: AVH alleviation**							
Dellazizzo et al., 2020 [38] Canada	Single-arm study with 3-month follow-up	10 SZ/SZA with AVH (M 43.4, SD 14.6, male 80%); N = 10	Samsung GearVR HMD set; avatar face (Morph3D character system) and voice (Roland AIRA VT-3), BehaVR software with Unity game engine	CBT for AVH included goal-setting and learning about AVH, diverse attributional mechanisms, mindfulness exercises, use of alternative explanation and relapse prevention, etc. VRT included one session for creating an avatar and eight sessions for therapy.	18 by combining CBT + VRT (9 individual, weekly, 1 h sessions with either CBT for AVH or VRT)	BAVQ-R, PSYRATS-AH, PANSS, BDI-II, Q-LES-Q-SF	Auditory verbal hallucinations, depressive symptoms, schizophrenia symptoms (both negative and positive), beliefs about voices, and QoL all showed significant improvement. Effects of CBT + VRT on symptoms of schizophrenia and depression were greater than those demonstrated by either intervention alone. FU: 3-month FU on CBT (T3) and 3-month FU on VRT (T5); most gains were maintained or further improved at T5, but beliefs about voices at T5 retreated closer to baseline level.
Dellazizzo et al., 2021 [39] Canada	Randomized parallel comparative pilot trial with follow-ups at 3, 6, and 12 months	37 VRT (M 43.6, SD 12, male 78.4%) and 37 CBT (M 41.4, SD 13.4, male 73%); N = 74 (11 dropouts)	Same as Dellazizzo et al., 2020 [36]		9 (individual weekly 1 h sessions with either CBT for AVH and VRT)	PSYRATS, BAVQ-R; secondary outcomes: BDI-II, PANSS, Q-LES-Q-SF, semi-structured interview	Both interventions resulted in substantial reductions in the severity of AVH and depressive symptoms. While VRT did not statistically significantly outperform CBT for AVH, it did have a greater impact on AVH overall and on affective symptoms. A significant impact from VRT was also seen in QoL and persecutory beliefs. FU: 3, 6, and 12 months; effects maintained for up to 1 year
du Sert et al., 2018 [40] Spain	Randomized, partial cross-over pilot trial, with a follow-up of 3 months	7 VRT and 7 TAU (TAU received a delayed 7 weeks of VRT) (M 42.9, SD 12.4, male 66.7%); N = 19 SZ with AVH (4 dropouts)	Samsung GearVR HMD set, avatar face (Morph3D character system) and voice (Roland AIRA VT-3), BehaVR software	VRT consisted of one avatar-creation session and eight therapeutic sessions with each containing pre-immersion, immersion, and post-immersion debriefing.	7 (weekly sessions including one avatar-creation session and six sessions of 45 min therapy).	PSYRATS, BAVQ-R, PANSS, BDI-II, QLESQ-SF, fear and anxiety scale (after each VRT)	Substantial improvements in AVH, depressive symptoms, and QoL were achieved with VRT. VRT had notably strong therapeutic benefits on the distress brought on by the voices. Participants gave high credibility to their avatars, feeling as though their persecutors were present there. FU: 3 months; improvements remained significantly
**Intervention: paranoia/delusion reduction**							
Freeman et al., 2016 [41] UK	RCT	15 VR with cognitive therapy (M 42.1, SD 13.4, male 67%) and 15 VR (M 40.6, SD14.4, male 40%); N= 30 PPD	nVisor SX111 HDM 1280 × 1024, 60 hz refresh rate, a computer and tracking system (Intersense)	Virtual reality cognitive therapy; patients were exposed to two VR places (train and lift), gradually building up with more avatars each time. Total of 7 VR scenarios, 5 min each.	1 (~60–90 min in VR lab)	PANSS, PSYRATS-DS, SBQ-PB, BAI, BDI, VAS	Large reductions in delusional conviction (a 22% reduction) were seen in the VR experimental group, and they also showed a 19.6% reduction of distress in the real world. VR-based cognitive therapy proved to be efficient in reducing delusions.
Geraets et al., 2020 [8] Netherlands	RCT with 6-month follow-up	43 VR-CBT (M 38, SD 10, male 67.4%) and 48 TAU (M 40.9, SD 10, male 70.8%); N = 91 PP	Logitech F310 Gamepad, Vizard software, Sony HMZ-T1/T2/T3 HMD, 1280 × 720, 51·6 FOV, and a 3DOF tracker.	VR-CBT; the first two sessions presented the VR system and set personal goals. Remaining sessions consisted of 40 min practicing time in VR (a street, bus, café, and supermarket) and 20 min to plan and reflect on exercises.	16 (1 h one-on-one therapy sessions)	ESM by PsyMate	Subjects who participated in VR-CBT, compared to those in TAU, saw greater improvement in paranoia level (feeling hurt and disliked) and negative affect (insecure and down), but not in positive affect. Treatment had no effect on the way that emotional states and paranoia interacted. FU: 6 months; the change in paranoia and negative affect were maintained or further enhanced at FU
Pot-Kolder et al., 2018 [29] Netherlands	Single blind RCT with 6-month follow-up	58 VR-CBT group (M 36.5, SD 10, male 69%) and 58 TAU (M 39.5, SD 10, male 72%); N = 116 PPD (13 dropouts)	Logitech F310 Gamepad, Vizard software, Sony HMZ-T1/T2/T3 HMD, 1280 × 720, 51·6 FOV, and a 3DOF tracker	VR-CBT; to match the patient’s paranoid fears, therapists could change the avatars’ attributes (including sex and ethnicity), as well as their number (0–40) and how they responded to the patient	16 (one-hour individual therapy sessions).	Primary: time spent with others, ESM by PsyMate. Secondary: SBQ-PD, GPTS, SIAC, BDI, SOFAS, MANSA, DACOBS, ISMI, BCSS, BARS, IPQ, SSQ	Momentary anxiety and paranoid ideation were dramatically decreased in the VR-CBT group. However, the time spent with others (i.e., social participation) did not considerably increase after VR-CBT. Social cognition issue and a decrease in safety behavior were found to be the mediators of a shift in paranoid ideation. FU: 6 months; improvement maintained with further gains in social functioning and self-stigmatization
Pot-Kolder et al., 2020 [28] Netherlands	Single blind RCT with 6-month follow-up	58 VR-CBT (M 36.5, SD 10, male 69%) and 58 TAU (M 39.5, SD 10, male 72%); N = 116 PPD (13 dropouts)	Same as Pot-Kolder et al. 2018 [27]			Outcome measure: QALY (by GPTS) and social participation. Cost measures: (1) direct medical cost + VR costs; (2) direct travel costs; (3) indirect cost from productivity loss.	Between-group comparison showed statistically significant differences on all measures except monetary anxiety and indirect cost of productivity. The ICER for VR-CBT treatment was €48,868 per QALY gained compared to the €80,000 willingness to pay. Offering VR-CBT to patients with paranoid delusions is a financially viable strategy for cost-effectively enhancing patients’ health. FU: 6 months; there were 68 hospitalization days for TAU vs no psychiatric admission for VR-CBT
**Intervention: social stress & relaxation**							
Freeman et al., 2022 [42] UK	Parallel-group, single-blind RCT with 6-month follow-up	174 gameChange (M 36.6, SD 12.8, male 67%) and 172 TAU (M 37.8, SD 12.2, male 67%); N = 346 SSD with agoraphobic (9 dropouts)	Standalone VR headset	gameChange: designed to specifically target everyday situational anxiety. Six situations, including leaving the house, being in a café, shop, doctor’s office, or pub, as well as boarding a bus, in which patients practice various tasks. Each scenario had five levels of difficulty.	6 (over 6 weeks, each including 30 min in VR)	Primary: O-AS, O-BAT. Secondary: AMI-AS, C-SSRS, GPTS, PWQ, PHQ-9, EQ-5D, ReQoL, O-CDQ, BHS, BESAA	Agoraphobic avoidance and distress in everyday situations significantly decreased in the gameChange VR therapy group. Most secondary outcomes showed no significant changes, and the overall treatment effect sizes were small. However, the benefits of treatment increased with the degree of anxious fears and avoidance. FU: at 6-month FU, moderate-to-large improvements in agoraphobic avoidance were maintained
Rault et al., 2022 [43] France	Single-arm pilot study	13 SZ/SZA (M 43, SD 11.5, male 77%); N = 13	HMD (Oculus Rift) with a 360° view	Relaxation therapy used “C2 Hypno” category, which included 4 pre-selected landscapes in VR	5 (30 min duration for each session with 10 min of VR exposure)	PANSS, ITQ, CDS, SSQ, VASA, CAS	Anxiolytic effects were demonstrated in the COVI and VAS scales. The VR relaxation therapy was not aimed to improve psychotic symptoms, but the PANSS tended to improve.
Tan et al., 2021 [12] Singapore	Single-blinded pilot RCT	19 VR relaxation (male 26.3%) and 20 WL (male 52.4%); N = 40 PP/BD/SZ	HMD (iTVGoggles Wide View 3D+), videos developed by Klainin-Yobas et al. (2015)	V-DESSERTS; comprised psychoeducation (20 min) and virtual screen-based relaxation practice (20 min). WL were brought into the same quiet room for 40 min to read health pamphlets	Twice-daily individual-based sessions	PSS, NSRS, HR, BP and ST, PRS, KSMMQ	The intervention group reported significantly increased levels of perceived relaxation and knowledge, but results on subjective and objective stress were inconclusive.
Veling et al., 2021 [44] Netherlands	Crossover RCT	25 VRelax and 25 standard relaxation; then crossover to other group after 10 days (M 41.6, SD 14.2, male 34%); N = 50 BD/PP (1 dropout)	Samsung Gear VR set with the 360° videos developed by The Dolphin Swim Club, VIEMR, and Atmospheres	VRelax used immersive 360° videos of nature with some added interactive components. Subjectss were free to choose the video within the app at home. Standard relaxation subjects used audio tracks of progressive relaxation exercises and guided meditation	10 (at least once daily for 10 min during a 10-day period)	Primary: VAS. Secondary: PSS, IDS-SR, BAI, GPTS, SSQ	Based on the improvements in all VAS categories, both groups reported a statistically significant decrease in negative affective states and an increase in positive affective states. Compared to relaxation exercise, VRelax had a greater positive impact on negative affective states, especially when it came to feeling down, cheerful, or anxious. Ten-day measurements of psychiatric symptoms showed some improvement in both conditions.

Abbreviations: AI, assertion inventory; AMI-AS, Agoraphobia Mobility Inventory Avoidance Scale; AVH, auditory voice hallucination; BAI, Beck Anxiety Inventory; BARS, Brief Adherence Rating Scale; BAVQ-R, Beliefs About Voice Questionnaire—Revised; BCIS, Beck Cognitive Insight Scale; BCMET, Baron-Cohen Minds in the Eyes Test; BCSS, Brief Core Schema Scales; BDI-II, Beck Depression Inventory-II; BESAA, Body-Esteem Scale for Adolescents and Adults; BHS, Beck Hopelessness Scale; BP, blood pressure; CAARMS, Comprehensive Assessment of the At Risk Mental State; CAPE, Community Assessment of Psychic Experiences; CASH, Comprehensive Assessment of Symptoms and History; CAVIR, Cognition Assessment in Virtual Reality; CBT, Cognitive–Behavioral Therapy; CDS, Cambridge Depersonalization Scale—State Version; CDSS, Calgary Depression Scale for Schizophrenia; CPT, Continuous Performance Test; CSSRC, Columbia Suicide Severity Rating Scale; CT, controlled trial; CTQ-SF, Childhood Trauma Questionnaire—Short-Form; DACOBS, Davos Assessment of Cognitive Biases Scale; DART, Danish Adult Reading Test; DOG, Dogmatism Scale; DS, digit Span; EQ-5D, EuroQol Questionnaire—5D, ESM, experience sampling method; FAST, Functional Analysis Screening Tool; FEP, first-episode psychosis; FOV, field of view; GAF, Global Assessment of Functioning; GPTS, Green Paranoid Thought Scale; GSR, galvanic skin response; HC, healthy control; HDM, head-mounted display; HDRS-17, Hamilton Depression Rating Scale; HR, heart rate; HF, high frequency; IPQ, Igroup Presence Questionnaire; IP, integrated psychological treatment; ISMI, Internalized Stigma of Mental Illness Questionnaire; KSMMQ, Knowledge on Stress and Medication Management Questionnaire; LF/HF, low-frequency to high-frequency ratio; LQoLP, Lancashire Quality of Life Profile; M, mean age; MANSA, Manchester Short Assessment of Quality of Life; MD, mood disorder; MINI, Mini International Neuropsychiatric Interview; MNSE, Mini Mental State Examination; MWT-B, Multiple-Choice Vocabulary Intelligence Test; O-AS, Oxford Agoraphobic Avoidance Scale; O-BAT, Oxford Behaviour Avoidance Test; O-CDQ, Cognitions and Defences Questionnaire; OTS, One-Touch Stocking of Cambridge; PANSS-DS, Positive and Negative Syndrome Scale—Delusion Scale; PHQ-9, Patient Health Questionnaire 9; PP, patients with psychosis; PPD, patients with persecutory delusions; PQ, Presence Questionnaire; PRS, perceived relaxation scale; PSD, psychosis spectrum disorders; PSS, perceived stress scale; PST, processing speed test; PSYRATS-DS, Psychotic Symptom Rating Scales—Delusion Scale; PWQ, Paranoia Worries Questionnaire; Q-LES-Q-SF, Quality of Life Enjoyment and Satisfaction Questionnaire—Short-Form; RAVLT, Rey Auditory Verbal Learning Test; RBANS, Repeatable Battery for the Assessment of Neuropsychological Status; RCT, randomized controlled trial; ReQoL, Recovering Quality of Life Questionnaire; ROP, recent-onset psychosis; RPM, Raven’s Progressive Matrices; RVP, rapid visual information processing; RV-REF, Virtual Reality Program for Emotional Recognition; SADS, social avoidance and distress scale; SANS, Scale for the Assessment of Negative Symptoms; SAPS, Scale for the Assessment of Positive Symptoms; SBQ-PB, Safety Behaviours Questionnaire—Persecutory Beliefs; SBQ-PD, Safety Behaviours Questionnaire—Persecutory Delusions; SCAN, Schedules for Clinical Assessment in Neuropsychiatry; SCIP, Cognitive Impairment in Psychiatry; SCL, skin conductance level; SCWT, Stroop Color and Word Test; SD, standard deviation; SERS, Self-Esteem Rating Scale; SFS, social functioning scale; SIAS, social interaction anxiety scale; SOFAS, Social and Occupational Functioning Assessment Scale; SPP, siblings of psychotic patients; SSIT, simulated social interaction test; SSPS, State Social Paranoia Scale; SSQ, Simulator Sickness Questionnaire; ST, skin temperature; SUD, momentary subjective distress; SUS, System Usability Scale Questionnaire; SWM, spatial working memory; SZ, schizophrenia; SZA, schizoaffective; TMT, Trail-Making Test; UPSA-B, UCSD Performance-Based Skills Assessment; VAS, Visual Analog Anxiety Scale; V-DESSERTS, virtual screen-based stress management program; VFT, verbal fluency test; VLMT, Verbal Affective Memory Test; VLT-I, Verbal Learning Test—Immediate; VR, virtual reality; VREQ, Virtual Reality Experience Questionnaire; VRSSQ, VR Simulation Sickness Questionnaire; VRT, VR-assisted therapy or avatar therapy; VR-ToMIS, VR-assisted Theory of Mind intervention; WAIS, Wechsler Adult Intelligence Scale; WCST, Wisconsin Card Sorting Test; WL, waitlist; WMT, working memory test; YMRS, Young Mania Rating Scale.

### 3.2. Sample Size, Participants and Study Design

The sample sizes varied, ranging from 10 to 346 participants (mean = 81 and median = 48). Still, most had relatively small sample numbers, with half of the studies having fewer than 50 subjects. The participants in the studies were diagnosed with recent onset or first-episode psychosis (FEP) and non-affective psychotic disorders including schizophrenia and schizoaffective disorders. Some studies focused on non-affective psychosis patients who were suffering from paranoid ideation and treatment-resistant refractory AVH.

Regarding the research design, there were thirteen pilot studies, six non-pilot RCT, one validation study, one single-arm study, and seven cross-sectional, between-subject design studies. Approximately 46% of the papers being pilot studies in this review, which may explain why the sample sizes tended to be smaller.

### 3.3. VR Equipment and System Used

The types of equipment used by the studies have different specifications, which may produce different senses of immersion. Among them, three studies did not provide details of the HMD that was used [34,35,36]. Three publications used 3D glasses [26,27], with one specifying polarized glasses for watching a 3D stereoscopic projection provided by two multimedia projectors. This setup is closer to artificial reality (AR) [33].

For those that specified the details of the HMD, brand models included Sony HMZ-T1/T2/T3 (8 studies), Samsung Gear VR (5 studies), eMargin Z800 3DVisor (3 studies), Oculus Rift (2 studies), Oculus Go (1 study), nVisor SX111 (1 study), iTVGoggles Wide View 3D+ (1 study), and standalone HDM by Oxford VR (1 study).

VR headsets could be divided into three categories: PC-powered, all-in-one (also called standalone), and wearable smartphone holders. Samsung Gear VR belongs to the third type, as a smartphone must be snapped into the gear to display the scenes. It is easy-to-carry and low-cost but has limited features. Oculus Go and Oxford VR are standalone HDMs with the latest technology; they contain display, control, and storage functions and can be operated without any external support. iTVGoggles Wide View 3D+ is also an all-in-one HMD, but its function is relatively simple and is largely for watching 3D videos. The rest of the HDMs were PC-powered VR products that reliy on external computing devices such as PCs, gaming consoles, and other devices for control and storage.

Some papers also mentioned details of the software used to produce their own VR environment, while others directly adopted finished products from 3D video suppliers. These included CleVR with Vizard software and Logitech Gamepad (10 studies), Unity 3D game engine (3 studies), NeuroVr2.0 software (2 studies), Vtime (1 study), C2 Care (for C2Hypno) (1 study), and VIEMR, The Dolphin Swim Club, and Atmospheres for 3D videos (1 study).

### 3.4. VR Environments and Tasks

Most studies used VR to simulate the daily life environments that were more engaging to the participants; some example settings include street, bus, metro, café, bar, supermarket, lift, beach, campground, valley, park, open-plan office, kitchen or room of a house, etc. For relaxation studies, the VR environments simulated different natural landscapes.

In order to assess cognitive and social functioning, participants were required to conduct different tasks using VR, such as recollecting items (object task) and faces (social task) [30], recognizing facial emotions of the avatars [33], sitting at a table with the other two avatars to have conversations [31], or preparing a meal in a simulated kitchen [32].

In order to assess the relationship between paranoid ideation and social stress, participants were required to carry out duties in an open-plan office with positive or negative feedback from their avatar colleagues [34] or visit a café or bar with three variations [13,20,21,22,23,24,25]: number of avatars (from 0 to 40), ethnic density (80% or 20% of their own ethnic avatars), and levels of hostility (angry or neutral facial expression of the avatar).

For cognitive training, participants were asked to carry out hierarchical sequences of tasks in four different virtual environments, including park, valley, beach, and supermarket [35,36]. For social training, the participants practiced social interactions with one or more virtual avatars in daily life settings and were encouraged to gradually build up their social skills repertoire [26,27]. For Theory of Mind (VR-ToM) training, each VR session was based on three consecutive steps: (1) engaging in simulated social interactions for a conversation with an avatar, (2) being assigned a task to interpret the avatars’ implied emotions, and (3) discussion with a trained psychotherapist who applied cognitive and metacognitive techniques [37].

To alleviate the impact of AVH, schizophrenia patients were offered VR-assisted therapy or avatar therapy (VRT) on top of conventional cognitive–behavioral therapy (CBT) [38,39,40]. VRT consisted of creating an avatar of their persecutor and having conversations with the created avatar, which allowed the patients to confront the reproduced hallucination experience. Each session of VRT was designed to progress in three phases: (1) pre-immersion (discussion with the therapist), (2) immersion (entering into a dialogue with their avatar), and (3) post-immersion (debriefing and evaluating their feelings).

To reduce paranoid ideation or persecutory delusions, patients with psychotic disorders were provided with graded exposure to the virtual social environments that elicited fear, paranoid thoughts, and safety behaviors. Three out of four studies used VR-based individualized CBT therapy (or VR-CBT) as an intervention [8,28,29]. The number of avatars (0–40), the characteristics of the avatars (including sex and ethnicity), and the avatars’ responses to the patient (neutral or hostile, eye contact) varied to match the paranoid fears of the patients.

For stress relaxation intervention, the participants practiced relaxation by watching nature videos with or without audio guidance, and some videos had interactive elements to increase enjoyment [12,42,43,44].

### 3.5. Duration and Structure of the VR-Based Assessment or Intervention

Ten of the twelve assessment studies conducted the experiments in a single session. One study consisted of three sessions [34], and another consisted of four sessions [30]; both of these studies contained VR exposure in only two of these sessions.

In general, there was wide variation in the structure of the therapies across the VR-based intervention studies, with a greater number of sessions ranging from 5 to 18 sessions (Table 2 for details), except for two studies. The RCT conducted by Freeman et al. (2016) required the participants to attend one session only, which lasted about 60–90 min in the VR laboratory [41]. Another pilot RCT led by Tan et al. (2021) consisted of two individual sessions on the same day, each lasting 40 min [12]. For the rest of the studies, the length of each session varied from 10 to 90 min. Interventions based on cognitive therapy usually lasted for 45 min to an hour per session, similar to the conventional sessions.

### 3.6. Measurements

Table 3 summarizes the clinical, functional, and cognitive assessments used in different studies. The number of standardized assessments for each study ranged from 1 to 15, with a mean of 5.8 per article. Furthermore, three of them reported the use of more than 10 measurement instruments in the experiment [32,34,42]. These instruments covered different areas including diagnosis, psychopathology, neuropsychiatric or cognitive functioning, mood, paranoid or delusional ideation, social anxiety or distress, social and global functioning, quality of life, VR experience and self-concept, etc.

### 3.7. Follow-Up and Dropout of Intervention Studies

Eight out of the sixteen intervention studies conducted follow-ups. Most were 3-, 4-, or 6-month follow-ups; only one study performed a 1-year follow-up [39]. Most of the gains or effects were largely maintained, and in some cases, there were additional improvements or further enhancements on affect, social functioning, and self-stigmatization [8,29].

Seven studies explicitly indicated the dropout numbers, ranging from 2% to 21% dropout rates, with a mean of 14%. Hesse et al. (2017) reported that the reasons for dropout were simulator sickness and feelings of overstimulation [34]. The authors also suggested that the design of three weekly sessions for assessment tests might have caused higher dropout than a single-session design would have. Dellazizzo et al. (2021) [39] explained that the dropout rates, while slightly larger for VRT, were in a similar range as other psychosocial interventions. Du Sert et al. (2018) revealed that dropout was due to anxiety after the first therapeutic session and a lack of engagement with the therapy model [40]. In Pot-Kolder et al. (2018), the eleven dropout participants included four who never started treatment [29]. The remaining seven patients discontinued for reasons such as being too afraid, having no time, being unwilling to travel, feeling nausea, or the HMD being too uncomfortable to tolerate.

### 3.8. Validity of Using VR as an Assessment Tool

#### 3.8.1. Cognitive and Social Functioning

Two of the four studies that used VR to measure one’s cognitive and social functioning suggested that this application was useful. In Han et al. (2014), patients with schizophrenia had significantly more active avoidance of eye contact during virtual three-party conversations in the speaking phase, and patients also took longer to start speaking than healthy controls [31]. Miskowiak et al. (2022) used CAVIR, a VR assessment tool that assessed verbal memory, processing speed, attention, working memory, and planning skills in an interactive virtual reality kitchen scenario in which the participants were asked to prepare a meal. The performance of the patents in CAVIR demonstrated strong correlations with other neuropsychological tests, which proved CAVIR to be a sensitive and valid instrument [32].

However, Dietrichkeit et al. (2020) reported that the memory tests under VR environments could only partially distinguish the patient group from the healthy control group, as their performance only differed significantly on the social task (recalling faces) but not the object task (recalling items) [30]. The tasks intended to assess the participants’ memory bias. Moreover, there was no difference between patients and healthy controls in terms of emotional recognition as assessed using the VR program called RV-REF [33].

#### 3.8.2. Social Stress and Paranoid Ideation

Studies found that both the patient and the healthy control groups who were exposed to social stressors (e.g., population density, ethnicity, and hostility) in a VR environment had increased autonomic stress responses such as heart rates, high frequency, low-frequency to high-frequency ratio, and skin conductance level [21] and increased their interpersonal distance [22]. Furthermore, in both patient and control groups, studies found that the effect of social–environmental stressors on paranoid ideation was further enhanced by negative self-esteem [23], cognitive bias [20,24], and childhood trauma [25]. Among different social stressors, only population density (i.e., the number of avatars) had a strong positive effect on both paranoia and distress, while hostility and ethnicity did not [13].

Findings were more varied in patients and the healthy population regarding the impact of VR-induced social stress on their physiological and psychological reactions. For instance, Counotte et al. (2017) reported that galvanic skin response in patients, but not in healthy controls, was significantly stronger in virtual environments with avatars of another ethnicity [21]. In another study, patients demonstrated statistically heightened paranoid ideations after social rejection in a VR open-plan office, but it was not different among healthy controls [34].

### 3.9. Effectiveness of Using VR as an Intervention

#### 3.9.1. Cognitive and Social Skill Training

Of the five studies that used VR as an intervention tool to improve cognitive performance and social skills in psychotic disorders, all found significant improvement following the intervention. VR-CRT showed additional improvements in planning and divided attention and fewer cognitive deficiencies in schizophrenia patients compared to the control group that received integrated psychological treatment (IPT), which also helped to enhance sustained attention [35,36]. After taking the VR-based social training program called Soskitrain, patients with schizophrenia or schizoaffective disorder exhibited significant improvements in psychopathology, negative symptoms, social anxiety, avoidance, and social skills mastery. More encouragingly, the study found that Soskitrain also contributed to the patients’ generalization of newly learned skills into their daily functioning [26,27]. Moreover, with the VR-ToMIS program, the experimental group, which was placed in a social VR environment and then debriefed by a psychotherapist on applying cognitive and meta-cognitive techniques, reported moderate to large therapeutic effects in improving negative symptoms, immediate memory, ToM and pragmatic language skills, etc. in a randomized pilot study involving schizophrenia outpatients [37].

#### 3.9.2. AVH Alleviation

VRT that externalized hallucinations through the creation of an avatar was shown to effectively improve AVH severity, depressive symptoms, and quality of life in a randomized partial cross-over pilot trial involving schizophrenia patients [40]. A later randomized pilot study [39] compared VRT with CBT in reducing AVH in patients with treatment-resistant schizophrenia or schizoaffective disorder, and the results did not indicate that VRT produced a significant superior therapeutic effect over conventional CBT for AVH. Nonetheless, VRT did achieve larger effects, particularly in reducing overall AVH and affective symptoms, and showed significant results on persecutory beliefs and quality of life. Another pilot single-arm study combined the use of both VRT and CBT interventions; the improvements in depressive and psychotic symptoms for patients with schizophrenia or schizoaffective disorder were larger than those reported by either intervention alone [38].

#### 3.9.3. Paranoia/Delusion Reduction

Of the four studies that used VR to treat paranoid ideation, all of them adopted either cognitive therapy or CBT as intervention. In Freeman et al. (2016) [41], psychosis patients with persecutory delusions from the VR cognitive therapy group reported larger reductions in delusional conviction and transference of their reduction of distress into the real world than the VR exposure group, which was merely exposed to VR without receiving cognitive therapy. Geraets et al. (2020) [8] and Pot-Kolder et al. (2018) [29] demonstrated that VR-CBT significantly improved momentary paranoid ideation, momentary anxiety, and negative affect in psychotic patients but did not increase social participation with other people. Not only did the VR-CBT prove to be highly effective in treating paranoid delusions, but Pot-Kolder et al. (2020) [28] showed it was also an economically viable approach after conducting a cost-effectiveness analysis (CEA) and calculating ICER from a societal perspective.

#### 3.9.4. Social Stress and Relaxation

The four studies that tested the effectiveness of VR-based intervention and relaxation programs all generated positive results. The gameChange VR therapy yielded significantly reduced agoraphobic avoidance and distress in everyday situations among severely anxious patients with schizophrenia spectrum disorder [42]. VR relaxation therapy reduced anxiolytic effects and improved the PANSS scores of patients with schizophrenia or schizoaffective disorder [43]. The patients who completed V-DESSERTS intervention reported significantly higher perceived relaxation and knowledge, but there were inconclusive results on subjective and objective stress reduction [12]. Compared to standardized relaxation exercise, VRelax produced a statistically significant reduction in negative affective states and improved positive affective states as well as psychiatric symptoms [44].

### 3.10. Feasibility or Acceptance of the Use of VR

There were twelve studies that used quantitative studies with standardized assessments (Table 3) or qualitative interviews to collect participant feedback on their VR experiences. Most of them reported a strong sense of presence in the VR environments and minimal simulation sickness [29,31,32,43,44], and one study further suggested there was a strong correlation between real-world symptoms and those induced in VR [20]. The patients in Vass et al. (2020) [37] and Tan et al. (2020) [12] reported that VR tools were engaging, interesting, and safe to use. Participants in Rus-Calafell et al. (2014) [27] reported a high level of satisfaction with regard to the perceived intervention’s benefits and the psychologist’s work, as well as acceptance (non-aversive) of the use of VR. Other studies [26,39,43] also found good tolerance and acceptance of therapies using VR technology.

## 4. Discussion

### 4.1. Implications for VR-Based Assessments for Psychosis

This systematic review provided an overview of the application of immersive VR in the assessment and treatment for patients with psychotic disorders. We found that not all VR tasks in the assessment studies could distinguish between patients and healthy participants with respect to their physiological responses, paranoid ideation, and certain aspects of cognitive functioning such as memory bias on object tasks. This finding was consistent with that of another review on VR-assisted psychiatric assessments by Geraets et al. (2022) [45]. It was noted that most of these studies on VR assessments still collect data based on self-reported measures such as GPTS and SSPS, which could not take full advantage of using VR to track the participants’ responses objectively. Thus, most assessment studies included in our review seem more to prove the validity of using VR environments to stimulate changes in subjects, rather than as a valid tool for assessing the differences between the subject groups. Nonetheless, Miskowiak et al. validated the use of a performance-based VR program (CAVIR) for assessing cognitive functions in patients with psychosis spectrum disorders against those of existing neurocognitive test batteries [32]. In the CAVIR, patients were required to perform five tasks to prepare a meal in a simulated kitchen. Results showed a strong association between CAVIR and other neurocognitive tests, providing preliminary evidence on the concurrent validity of this VR-based cognitive assessment.

Evidence using VR as an assessment tool to differentiate the physiological reactions between psychotic patients and controls was mixed. Favorably, Souto et al. found that only patients had an alpha-frontal activity change under VR-based exposure to anger and disgust stimuli, not healthy participants [33]. However, other studies showed that VR-based assessment was less useful in differentiating physiological responses such as the change in heart rate and blood pressure in the two groups, although group differences were made in other responses such as eye gaze and galvanic skin response [20,31]. Since these bodily signs should be objective measures, it is worth investigating whether the mixed results were caused by measurement errors when the subjects move around during the VR tasks, or it was due to the ceiling effects: while VR might have caused more social stress in the patient group than in the control group, heart rate did not show a proportionate increase due to our physical limitations, so the difference between the two groups was less likely to be seen.

Though not reviewed in this study, self-agency and egocentric perception have also been assessed under non-immersive VR environment in schizophrenia patents and healthy controls. Studies found that when patients received no feedback, they demonstrated more impairments than controls in telling whether they were responsible for their pointing actions [46]. Another study found that compared to healthy controls, patients’ ability to take egocentric perspectives was intact (e.g., when asked to judge which object is closest to them); however, patients demonstrated more difficulties with other allocentric referencing conditions (e.g., when asked to judge which object is closest to a ball or closest to a palace) as well as switching between these allocentric referencing conditions [47]. Together, these studies provided insights as to how a different sense of self would impact the group-specific information processing under complex VR environments.

Notably, this review of assessment studies focused on how well these VR tasks could differentiate between patients and healthy participants but did not touch on its application for differentiating subjects with other subclinical conditions such as schizotypy and psychotic-like experiences. Accumulating evidence from physiological to clinical level supported the notion that psychosis exists on a continuum [48,49,50]. One study that adopted VR-based assessment for persecutory ideation succeeded in differentiating subjects between low paranoia, high nonclinical paranoia, and persecutory delusions, supporting the idea of a spectrum of paranoia in the general population [51]. The application of VR-based assessment to sub-syndromal or subclinical populations, on top of the clinical population, would be helpful in examining the validity of the continuum notion of psychosis.

### 4.2. Implications for VR-Based Interventions in Psychosis

On the other hand, our review demonstrated that VR-based psychosocial interventions are relatively more promising, with most studies showing positive evidence for improving cognitive impairments, social skills, AVH, paranoid ideation and persecutory delusions, agoraphobic avoidance, negative and positive affective states, and other psychiatric symptoms in patients.

Antipsychotic medication is still the mainstream treatment for patients with psychotic disorders, while VR-based intervention, similar to other psychosocial interventions, is an adjunct treatment option on top of medications. Notably, pure exposure to VR environments did not result in a significant treatment effect unless it was combined with principles from cognitive and behavioral therapies [39]. In fact, most interventions reviewed here blended VR with conventional evidence-based therapies such as cognitive rehabilitation therapy (CRT), CBT, and avatar therapy (AT). For example, two studies [39,44] compared VR-based therapies with conventional therapies such as CBT and standard relaxation exercise. While both VR and non-VR-based therapies improved AVH, depressive symptoms, and psychotic symptoms, VR-based therapy improved AVH and negative affects slightly more, which is encouraging. Further investigation into how VR could enhance the effectiveness of traditional psychosocial therapies may provide valuable input in optimizing existing ones or creating new VR-based therapies.

So far, no research has been conducted on VR-based interventions that directly target negative symptoms in schizophrenia. There were, however, two pilot studies that found improvement in negative symptoms following VR interventions for social skills [27] and Theory of Mind training [36]. Studies with RCT designs are needed to explore if VR could become an alternative treatment for negative symptoms in schizophrenia.

### 4.3. Limitations

Since our review included publications written in the English language with a subject target of patients with psychotic disorders, some interesting uses of VR that may have therapeutic effect could have been left out in this review. In addition, as the application of VR technology is still relatively new, with a certain portion of the research papers being pilot studies, the results of the studies may not be entirely conclusive. Moreover, the types of VR equipment used in the review were of a wide variety, given significant technological advancement over the last decade. The polarized glasses used in some studies, in fact, are properly a form of AR technology, which is similar to VR but is a slightly different concept, because AR combines both virtual and real-world settings, while VR, strictly speaking, is limited to fictional worlds. This may make the comparison between different studies less relevant. Finally, we note that some non-RCT studies that were identified as having a high risk of bias were included; we suggest caution when drawing conclusions from these studies.

### 4.4. Future Direction and Conclusions

As more global technology companies invest massively in VR, we expect more advanced HMD models and an increase in content development. Hardware specification improvement will continue for display performance, weight, viewing angle, motion sensing, tracking ability and control, etc. Newer equipment will enhance the VR experience by increasing immersiveness and reducing the feeling of nausea and other discomforts (dizziness, eye strain, headache, etc.), which in turn helps to lower the dropout rates in studies. Further, the rise in the number of software developers may help increase the amount of ready-made VR content to assist in the adoption of VR for psychosis. At the same time, it is possible to have a more sophisticated “storyline” in the therapy, a larger variety of difficulty levels for VR tasks, greater interactive or artificial intelligence (AI) elements and more complicated avatars, etc. By leveraging the latest technology developments, there are substantial opportunities to make future assessment and intervention tools more valid and effective and facilitate self-diagnosis, self-training, and personalization.

Overall, it is encouraging to see such favorable feedback from patients towards using VR technology and the initial promise of the positive treatment outcomes from these VR-based interventions. In addition, we expect that the disadvantages of using VR, including cybersickness, high costs, the bulkiness of the equipment, restricted choices in available VR design, and feelings of isolation [52] will be gradually removed with the advancement of technology. Thus, the future of VR-based assessment and intervention in psychosis looks innovatively promising. We suggest that both researchers and clinicians in the field to take advantage of the ongoing development of VR technology and continue designing better tools for assessing and treating patients with psychotic disorders.

## Figures and Tables

**Table 2:** Schedule and duration of different VR-based interventions for psychotic disorders.

Therapies	Studies	Session Schedule and Duration
VR Cognitive Rehabilitation Therapy	La Paglia et al., 2013 & 2016 [35,36]	10 weekly individual sessions of app. 90 min duration each, guided by a predefined protocol
Soskitrain	Rus-Calafell et al., 2013 & 2014 [26,27]	16 twice-weekly individual sessions over eight weeks of app. 60 min each, including two parts: (1) 30 min to discuss content and (2) reminding practice with the VR program
VR-ToMIS	Vass et al., 2021 [37]	9 individual weekly sessions of 50 min each, comprising 8 virtual simulation-based sessions and one pre-briefing session
CBT + VRT	Dellazizzo et al., 2020 & 2021 [38,39]	9 individual weekly one-hour sessions for CBT and VRT, separately (total of 18 sessions)
VRT	du Sert et al., 2018 [40]	7 individual weekly sessions, including one session for creating an avatar and six 45 min therapeutic sessions
VR-CBT	Geraets et al., 2020; Pot-Kolder et al., 2018 & 2020 [8,28,29]	16 one-hour individual therapy sessions, with each session spending 40 min on virtual program and 20 min on discussion with therapists
gameChange	Freeman et al., 2022 [42]	6 weekly sessions each involving 30 min of VR
VR relaxation therapy	Rault et al., 2014 [43]	5 sessions of 30 min each, including 10 min VR exposure followed by psychometric assessment
V-DESSERTS	Tan et al., 2021 [12]	2 sessions within the same day, each consisting of 20 min of psychoeducation and 20 min of virtual screen-based relaxation practice
VRelax	Veling et al., 2021 [44]	At least once daily for 10 min over a 10-day period

Abbreviations: CBT, cognitive–behavioral therapy; Soskitrain, VR-assisted Social Skills Training; V-DESSERTS, virtual screen-based stress management program; VR, virtual reality; VRelax, VR self-managed relaxation; VRT, VR-assisted therapy or avatar therapy; VR-ToMIS, VR-assisted Theory of Mind intervention.

**Table 3:** Measurements used in different studies.

Dimensions	No. of Studies	Standardized Psychometric Instruments
Diagnosis/symptoms	15	PANSS, PSYRATS, CAARMS, CAPE, SCAN, BAVQ-R, SAPS, SANS
Neuropsychiatric or cognitive functioning	16	MINI, RPM, BRANS, BCIS, MMSE, FAB, TMT-A/B, ToL, SCWT, WCST, VLMT (learning/recall), MWT B Vocabulary test, OTS, SWM, RVP, RAVLT, WAIS-III, SCIP, WMT, VLT-I/II, DS, VFT, PST, CPT II, ToM (BCMET, faux pas test and cartoon stories task, the Hungarian metaphor and irony test), DACOBS, DOG, CASH
Paranoia/delusion	13	GPTS, SSPS, CDS, SBQ-PD, C-SSRS, PWQ
Mood	8	CDSS, BDI, BAI, IDS-SR, BHS, HDRS-17, YMRS
Social anxiety/distress	12	IPD, SUD, SIAS, AI, SSIT, SADS, VASA, CAS, O-AS and O-BAT, AMI-AS, O-CDQ, PSS, NSRS
Global/daily functioning	5	FAST, UPSA-B, GAF, ESM
Social functioning	3	PSP, SFS, SOFAS
Quality of Life	6	LQoLP, Q-LES-Q-SF, MANSA, EQ-5D, ReQoL
VR experience	11	PQ, SSQ, IPQ, ITQ SUS
Self-concept	3	SERS, BCSS, BESAA

Abbreviations: AI, Assertion inventory; AMI-AS, Agoraphobia Mobility Inventory—Avoidance Scale; BAI, Beck Anxiety Inventory; BARS, Brief Adherence Rating Scale; BAVQ-R, Beliefs About Voice Questionnaire—Revised; BCIS, Beck Cognitive Insight Scale; BCMET, Baron-Cohen Minds in the Eyes Test; BCSS, Brief Core Schema Scales; BDI-II, Beck Depression Inventory—II; BESAA, Body-Esteem Scale for Adolescents and Adults; BHS, Beck Hopelessness Scale; CAARMS, Comprehensive Assessment of At Risk Mental States; CAPE, Community Assessment of Psychic Experiences; CASH, Comprehensive Assessment of Symptoms and History; CDS, Cambridge Depersonalization Scale—State Version; CDSS, Calgary Depression Scale for Schizophrenia; CPT, continuous performance test; CSSRC, Columbia Suicide Severity Rating Scale; CTQ-SF, Childhood Trauma Questionnaire—Short=Form; DACOBS, Davos Assessment of Cognitive Biases Scale; DART, Danish Adult Reading Test; DOG, Dogmatism Scale; DS, digit span; EQ-5D, EuroQol Questionnaire—5D; FAST, Functional Analysis Screening Tool; GAF, Global Assessment of Functioning; GPTS, Green Paranoid Thought Scale; HDRS-17, Hamilton Depression Rating Scale; IPQ, Igroup Presence Questionnaire; ISMI, Internalized Stigma of Mental Illness Questionnaire; SMMQ, Knowledge on Stress and Medication Management Questionnaire; LQoLP, Lancashire Quality of Life Profile; MANSA, Manchester Short Assessment of Quality of Life; MINI, Mini International Neuropsychiatric Interview; MNSE, Mini Mental State Examination; MWT-B, Multiple-Choice Vocabulary Intelligence Test; O-AS, Oxford Agoraphobic Avoidance Scale; O-BAT, Oxford Behaviour Avoidance Test; O-CDQ, Cognitions and Defences Questionnaire; OTS One-Touch Stocking of Cambridge; PANSS-DS, Positive and Negative Syndrome Scale—Delusion Scale; PHQ-9, Patient Health Questionnaire 9; PQ, Presence Questionnaire; PRS, perceived relaxation scale; PSS, perceived stress scale; PST, processing speed test; PSYRATS-DS, Psychotic Symptom Rating Scales—Delusion Scale; PWQ, Paranoia Worries Questionnaire; Q-LES-Q-SF, Quality of Life Enjoyment and Satisfaction Questionnaire—Short-Form; RAVLT, Rey Auditory Verbal Learning Test; RBANS, Repeatable Battery for the Assessment of Neuropsychological Status; ReQoL, Recovering Quality of Life Questionnaire; RPM, Raven’s Progressive Matrices; RVP, rapid visual information processing; SADS, social avoidance and distress scale; SANS, Scale for the Assessment of Negative Symptoms; SAPS, Scale for the Assessment of Positive Symptoms; SBQ-PB, Safety Behaviours Questionnaire—Persecutory Beliefs; SBQ-PD, Safety Behaviour Questionnaire—Persecutory Delusions; SCAN, Schedules for Clinical Assessment in Neuropsychiatry; SCIP, Cognitive Impairment in Psychiatry; SCL, skin conductance level; SCWT, Stroop Color and Word Test; SERS, Self-Esteem Rating Scale; SFS, social functioning scale; SIAS social interaction anxiety scale; SOFAS, Social and Occupational Functioning Assessment Scale; SSIT, simulated social interaction test; SSPS, State Social Paranoia Scale; SSQ, Simulator Sickness Questionnaire; SUD, momentary subjective distress; SUS, System Usability Scale Questionnaire; SWM, spatial working memory; TMT, Trail-Making Test; UPSA-B, UCSD Performance-Based Skills Assessment; VAS, visual analog anxiety scale; VFT, verbal fluency test; VLMT, Verbal Affective Memory Test; VLT-I, Verbal Learning Test—Immediate; VREQ, Virtual Reality Experience Questionnaire; VRSSQ, VR Simulation Sickness Questionnaire; WAIS, Wechsler Adult Intelligence Scale; WCST, Wisconsin Card Sorting Test; WMT, working memory test; YMRS Young Mania Rating Scale.

## Data Availability

Not applicable.

## Supplementary Materials

The following supporting information can be downloaded at https://www.mdpi.com/article/10.3390/brainsci13030471/s1: Supplementary Material on quality assessment for non-randomized and randomized studies.
