# Supplementary material for: Application of Immersive Virtual Reality for Assessment and Intervention in Psychosis: A Systematic Review
Source: Brain Sci. 2023 Mar 10;13(3):471. doi: 10.3390/brainsci13030471 (PMC10046161; doi:10.3390/brainsci13030471)
Supplement: Supplementary file 1 [file brainsci-13-00471-s001.zip › brainsci-2243077-supplementary.pdf]

Supplementary Material 1. Quality assessment for non-randomised and randomised studies.

A. Risk of bias assessment in non-randomised studies using ROBINS-I tools

	Confounding	Selection of participants into the study	Classification of interventions	Deviations from intended interventions	Missing data	Measurement of the outcome	Selection reported results	Overall
**Assessment studies**								
Dietrichkeit et al., 2020	Moderate	Low	Low	Moderate	Low	Low	Low	Low
Han et al., 2014	Moderate	Low	Low	Low	Low	Moderate	Low	Low
Miskowiak et al., 2022	Moderate	Low	Low	Low	Low	Low	Low	Low
Souto et al., 2013	Moderate	Low	Low	Moderate	Low	Moderate	Low	Moderate
Counotte et al., 2016	Moderate	Low	Moderate	Moderate	Moderate	Moderate	Low	Moderate
Geraets et al., 2018	Low	Low	Moderate	Moderate	Moderate	Moderate	Low	Moderate
Hesse et al., 2017	Low	Low	Low	Moderate	Serious	Low	Low	Moderate
Jongeneel et al., 2018	Moderate	Low	Moderate	Moderate	Low	Low	Low	Moderate
Pot-Kolder et al., 2017	Moderate	Low	Moderate	Moderate	Low	Low	Moderate	Moderate
Veling et al., 2014	Moderate	Low	Low	Low	Low	Moderate	Moderate	Moderate
Veling et al., 2016a	Moderate	Low	Moderate	Moderate	Low	Moderate	Low	Moderate
Veling et al., 2016b	Moderate	Low	Moderate	Low	Low	Moderate	Low	Moderate
**Intervention studies**								
La Paglia et al., 2013	Moderate	Low	Low	Low	Low	Low	Moderate	Low
La Paglia et al., 2016	Moderate	Low	Moderate	Low	Low	Low	Moderate	Moderate
Rus-Calafell et al., 2013	Moderate	Low	Moderate	Low	Moderate	Low	Low	Moderate
Rus-Calafell et al., 2014	Moderate	Low	Moderate	Low	Moderate	Moderate	Low	Moderate
Dellazizzo et al., 2020	Moderate	Low	Moderate	Low	Low	Low	Low	Moderate
Rault et al., 2022	Low	Low	Moderate	Moderate	Low	Moderate	Moderate	Moderate

B. Risk of bias assessment in randomised studies using the revised Cochrane risk-of-bias tool for randomized trials (version 2) (RoB 2)

	Randomisation process	Deviations from intended intervention	Missing outcome data	Measurement of the outcome	Selection reported results	Overall
**Intervention studies**						
Vass et al., 2020	~	✓	~	✓	~	~
Dellazizzo et al., 2021	~	~	X	✓	✓	~
du Sert et al., 2018	✓	X	X	✓	X	X
Freeman et al., 2016	✓	✓	✓	✓	✓	✓
Geraets et al., 2020	✓	~	✓	✓	✓	✓
Pot-Kolder et al., 2018	✓	~	~	✓	~	~
Pot-Kolder et al., 2020	✓	~	✓	✓	~	~
Freeman et al., 2022	✓	~	✓	✓	✓	✓
Tan et al., 2020	~	~	✓	✓	✓	~
Veling et al., 2021	✓	~	✓	✓	✓	✓

Risk levels are classified into: ✓ Low risk of bias; X High risk of bias; ~ Some concerns
